# Effects of Blank Quality on Press-Formed PEKK/Carbon Composite Parts

**DOI:** 10.3390/ma11071063

**Published:** 2018-06-23

**Authors:** Valentina Donadei, Francesca Lionetto, Michael Wielandt, Arnt Offringa, Alfonso Maffezzoli

**Affiliations:** 1 Department of Engineering for Innovation, University of Salento, via per Monteroni, 73100 Lecce, Italy; valentinadonadei@hotmail.it (V.D.); alfonso.maffezzoli@unisalento.it (A.M.); 2 GKN Fokker Technologies BV, 7903 AN Hoogeveen, The Netherlands; michael.wielandt@fokker.com (M.W.); arnt.offringa@fokker.com (A.O.)

**Keywords:** polymer matrix composites, laminates, thermoplastic matrix, PEKK, press forming, deconsolidation, stress relaxation, annealing

## Abstract

The causes of delamination and porosities during press forming of pre-consolidated flat laminates (blanks) made of carbon fiber-reinforced poly(ether ketone ketone) (PEKK) were addressed in this study. In particular, the quality of the blank laminate was investigated before and after infrared heating. The consolidation quality was evaluated by thickness measurements, non-destructive inspection (NDI), and optical microscopy. The experimental results confirmed that deconsolidation phenomena can be related to residual stresses formed during blank forming in an autoclave, then released during infrared heating (IR) of the blank, determining most of the defects in IR heated blanks. These defects, generated at the pre-heating stage, were not fully removed in the consolidation stage of the press forming process. An annealing treatment, performed on autoclave-consolidated blanks above the glass transition temperature of the matrix, was proposed to reduce the formation of defects during IR heating. The stress relaxation phenomena during annealing were modelled using a simple viscoelastic model.

## 1. Introduction

Composites are multifunctional materials combining the attractive features of different materials in order to obtain outstanding mechanical and physical properties which can be tailored to meet the requirements of a particular application. This design opportunity not possible with conventional materials explains the wide application range of composite materials in aerospace, marine, transportation, piping, and in the construction field for strengthening and thermal insulation [1,2,3,4,5].

In particular, continuous fiber-reinforced thermoplastic (CFRTP) composites are increasingly being used in automotive and aerospace industries for structural applications thanks to an increased toughness, a low level of moisture uptake, an easy welding ability, a high repair potential, and recycling possibilities [6,7]. Another key advantage of thermoplastic composite materials is given by their potential shorter processing time in comparison to thermosetting matrix composites due to the absence of the curing reaction of the matrix [8,9]. Thermoplastic composites can be formed and consolidated in a time scale of minutes by using low-cost manufacturing processes, such as stamping, welding, and co-consolidation [10,11,12,13,14]. As recently reported in the literature, stamping/press forming is a promising manufacturing process for high-performance thermoplastic composites [15].

**Figure 1:**
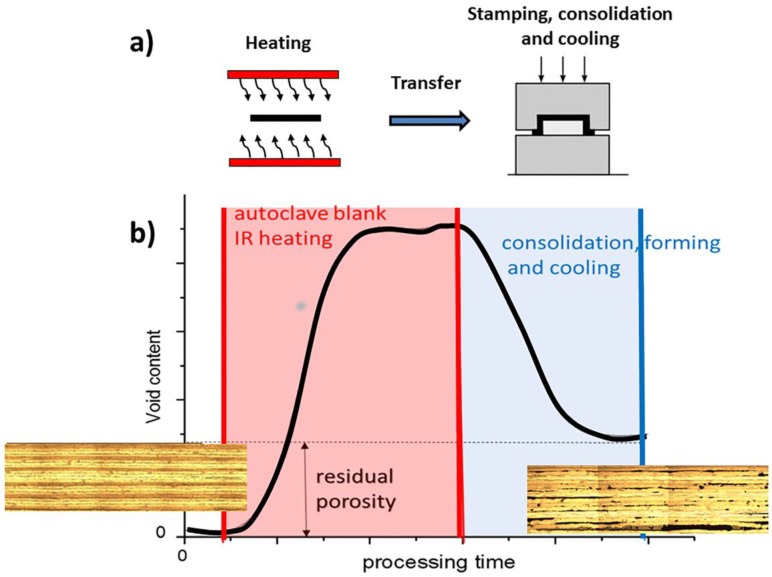
Schematic representation of the press forming process and the qualitative evolution of porosities during press forming.

In the press forming process, a pre-consolidated flat laminate, called blank, is preheated above the melting temperature of the polymer matrix in an oven, which is usually an infrared (IR) oven. Then, the blank is rapidly transferred to the preheated mold fixed on a press for the stamping operation, as schematically represented in Figure 1a. After consolidation and cooling under pressure, the formed component is demolded [16]. Blanks are rapidly heated in IR ovens, but their deconsolidation usually occurs due to the absence of an external compressive force during IR heating and blank transfer from the oven to the mold. Moreover, the component is cooled relatively quickly during the forming/consolidation stage and this can limit the re-consolidation process in the mold and the related reduction of defects generated during IR heating. Often, multiple heating cycles are needed first to consolidate a flat laminate blank, then to mold it and, eventually, local heating can be further required to weld it to other components. Each heating cycle partially erases the former stresses, but can also induce warpage and spring-in-angle phenomena [17]. Therefore, porosities, delamination, buckling, distortion, and warpage can be present in the final part with the inability of the component to retain the formed shape and a detrimental effect on its performance [18,19,20,21].

The possible development of the consolidation quality for an autoclave-consolidated blank made of a thermoplastic matrix composite is qualitatively shown in Figure 1b. The flat blank, consolidated in an autoclave, can be considered free of defects, as reported in the optical microscopy image on the left. After IR heating, many defects are detected and they can only be partially reduced in the matched die mold during press forming. Therefore, it is of essential importance to reduce or avoid the defects at the IR heating stage, since this currently limits the applicability of the press forming technology. The press forming process, mainly of carbon fiber-reinforced poly (ether ether ketone) (PEEK) or polyphenylene sulfide (PPS), has been widely studied for its high potential for a fast mass production [16,22,23,24]. However, a full comprehension of the causes of defects found in press-molded parts is not still available in the literature.

**Figure 2:**
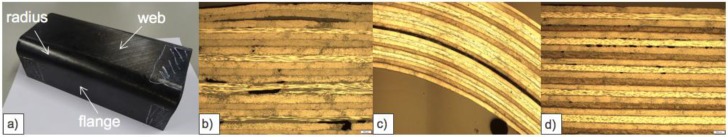
Typical defects in U shaped parts: (**a**) geometry of the product; (**b**) large voids in the flanges; (**c**) delamination in the web-radius; and (**d**) small porosities in multiple layers in the flanges.

This work studied some different possible causes of porosities and delamination at the heating stage of press forming of carbon fiber reinforced poly (ether ketone ketone) (CF/PEKK) composites. Several defects were detected on a typical press-molded U-shaped CF/PEKK part (Figure 2a), mainly in the web radius and flange zone, where a lower consolidation pressure was applied on the part during forming. In particular, large voids (Figure 2b) and small porosities in multiple layers (Figure 2d) were detected in the flanges and delamination in the web-radius (Figure 2c). These defects result from those formed during IR heating, the most critical step in the processing chain. The aim of this study was to investigate the causes of the blank deconsolidation during the IR heating: specifically, water and solvent evolution and the release of residual stresses were considered as the two phenomena possibly responsible for the generation of defects. The consolidation quality was evaluated by thickness measurements, non-destructive inspection, and optical microscopy. An annealing pre-treatment on autoclave-consolidated blanks was proposed in order to reduce delaminations during IR heating. Stress relaxation during annealing was studied and modelled using a simple viscoelastic model.

## 2. Materials and Methods

### 2.1. Materials

The material studied in this work was a pre-impregnated unidirectional tape supplied by Solvay (Alpharetta, GA, USA) with the trade name APC (PEKK-FC). It is a poly (etherketoneketone), reinforced with continuous AS4D 12K carbon fibers (66% by weight). The nominal thickness of the tape was 0.138 mm. As reported in the technical datasheet [25], the tapes were fully impregnated with a tailored fiber-matrix interface for optimal performance using a solvent impregnation technique. The glass transition temperature (Tg) and the melting temperature (Tm) of the PEKK matrix were, respectively, 159 °C and 337 °C, as reported in [25]. 

### 2.2. Sample Preparation and Heat Treatments

The blanks for press forming and deconsolidation studies were prepared by stacking 24 plies with a quasi-isotropic sequence [–45, 90, 45, 0]_3s_ and a nominal thickness of 3.3 mm, as reported in Table 1. They were fabricated in an autoclave at 6 bar, holding the laminate at 375 °C for 20 min, as recommended in the technical datasheet [25]. These blanks were cut in smaller specimens for stamp forming and deconsolidation tests were performed in an infrared (IR) oven (Watlow–Raymax (St. Louis, MO, USA) 1120 with a power of 3 W/cm^2^). Sample temperature was monitored until the hottest thermocouple reached 410 °C, usually in 220–240 s. Then, the samples were extracted from the oven and cooled in ambient air during a cycle of about 500 s. 

**Figure 3:**
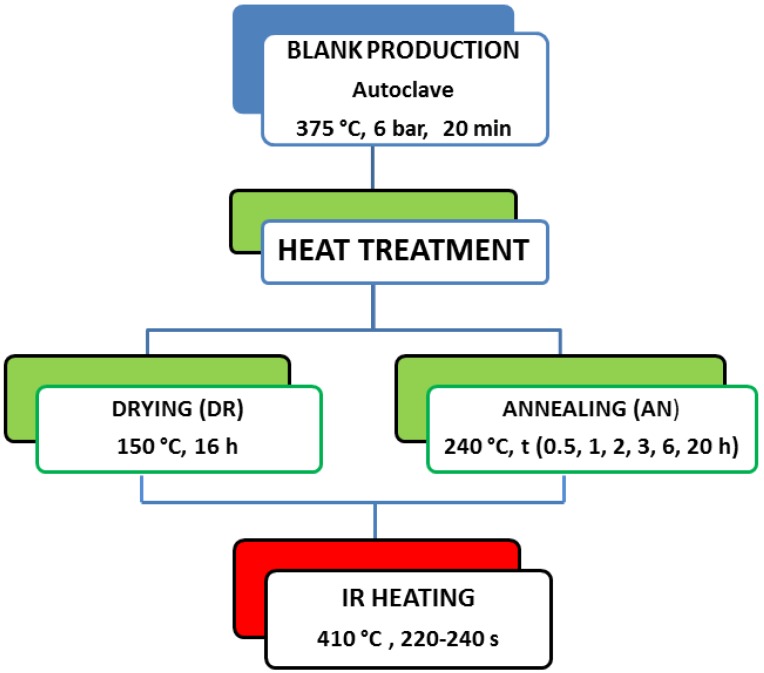
Schematic overview of the heat treatments performed on blank laminates.

As schematically reported in Figure 3, two different types of heat treatments were adopted before IR heating:

- a drying thermal cycle for 16 h at 150 °C, below Tg, devoted to moisture removal before IR heating;
- an annealing for 0.5, 1, 2, 3, 6, and 20 h at 240 °C, at a temperature above Tg in order to promote the relaxation of residual stresses developed during cooling in the autoclave, thanks to the mobility of the amorphous fraction of the matrix.

Moreover, the effectiveness of the annealing treatment on reducing the residual stresses was tested on non-symmetric laminate strips, with a [0, 0, 90] stacking sequence, as reported in Table 1. As is well known, any stack of anisotropic laminae led to the development of bending moments during cooling from the zero stress state, assumed at the consolidation temperature, when the matrix was transformed from liquid to a glassy polymer [26]. In fact, as known from lamination theory, the component B11 of the coupling stiffness matrix [B] was different from zero [26,27]. These moments aroused from residual stresses due to the anisotropic coefficient of thermal expansion of each unidirectional lamina. For the same reasons, symmetric laminates were also characterized by residual stresses arising from cooling, but they did not show any warpage. A strip obtained with only three plies [0, 0, 90] was consolidated in an autoclave at 375 °C and then cooled to room temperature. After autoclave consolidation, these samples were characterized by an evident bending due to the coupling between normal strains and bending moments.

### 2.3. Characterization

**Figure 4:**
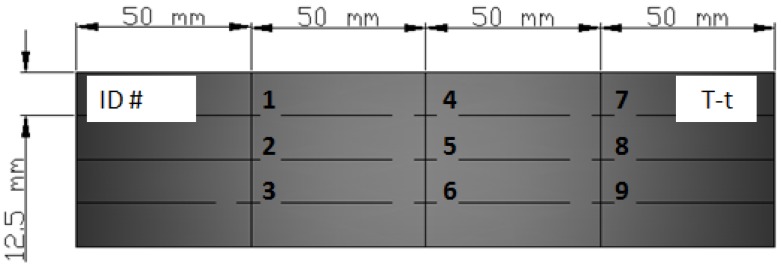
Measuring points of IR-heated laminates.

The IR-heated laminates were analyzed by thickness measurements at nine points (Figure 4) of the sample using a digital micrometer.

The curvature of the thin non-symmetric strips was evaluated by measuring the deflection in the center of the specimen, H, and the chord length of the specimen, L, using a digital micrometer.

Ultrasonic non-destructive inspection (NDI) was performed on samples after infrared heating by both A-scan and C-scan analyses. The A-scan analysis was performed with an ultrasonic probe with 12.5 mm diameter and 5 MHz central frequency, with an EPOCH 600 Digital Ultrasonic Flaw Detector (Olympus, Waltham, MA, USA) in the central area of the specimen to evaluate the effect of different pre-treatment methods on the deconsolidation quality. The C-scan analysis was performed on the samples using a 5 MHz ultrasonic probe and a scan speed of 500 mm/s using two water jets in single through-transmission mode. 

Porosities and delaminations were detected by optical microscopy on polished sections.

Thermogravimetric analysis (TGA) was performed to detect the possible presence of residual solvents used in the impregnation process or absorbed moisture. A METTLER TOLEDO STARe System (Schwerzenbach, Switzerland) SDTA851e was used for this analysis. Each sample was heated in air from 25 °C to 1000 °C at 10 °C/min. 

Table 1 summarizes the stacking sequence of the quasi-isotropic (QI) and non-symmetric (NS) composite samples investigated and the characterization methods used in this work.

## 3. Results

**Figure 5:**
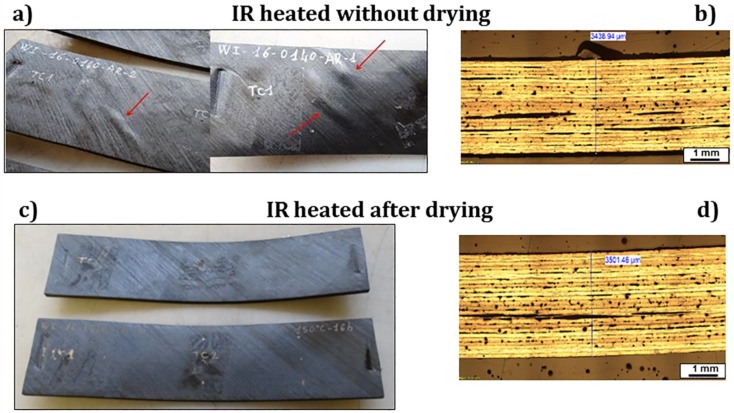
Defects of blank laminates after IR heating: (**a**) and (**b**) without drying; (**c**) and (**d**) after drying at 150 °C for 16 h.

After IR heating, the blank laminates not subjected to any heat treatment presented some macroscopic defects on the surface, like bulges, evidenced by the red arrows in Figure 5a, and delaminations and porosities, as evidenced by the optical microscopy image of Figure 5b. In order to reduce these defects, a drying cycle before IR heating was recommended by the prepreg manufacture since it was believed that water sorption during storage could cause delamination during IR heating. As indicated in Figure 5c,d, no macroscopic defects were detected on laminates dried at 150 °C for 16 h, even if warpage always occurred, probably as a consequence of the non-uniform fast heating and cooling to which these laminates were subjected. The effect of drying was, thus, to eliminate the bulges, but the porosities and delaminations still remained.

**Figure 6:**
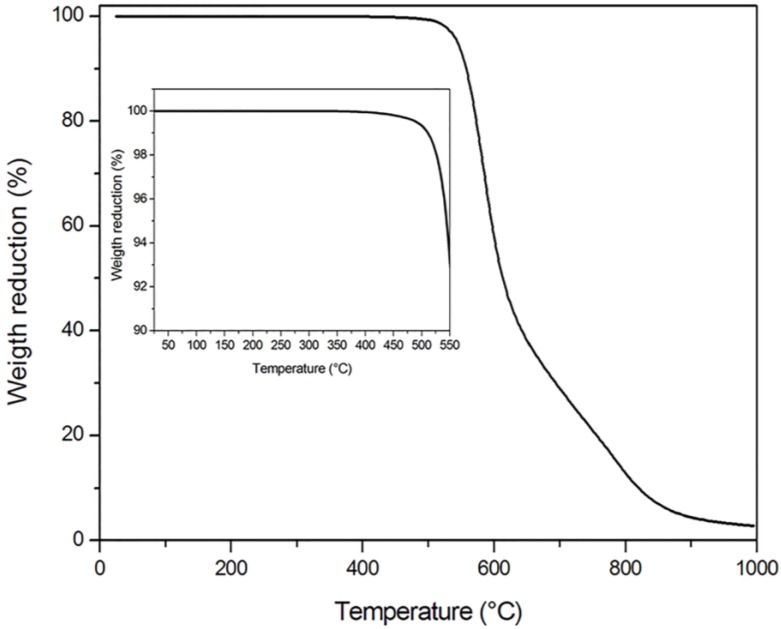
TGA thermogram in air on autoclave-consolidated blank composites.

Thermogravimetric analysis was then performed to detect the eventual presence of absorbed moisture or residual solvents used in the impregnation process. The TGA experiment on as-received CF/PEKK tape, not yet consolidated in the autoclave, detected limited weight losses below 180 °C, due to moisture evaporation, and above 340 °C. This latter value was attributed to the evaporation of residues of the high-boiling-point solvent used by the manufacturer during the impregnation process of the carbon fibers with the PEKK matrix. The TGA analysis on autoclave-consolidated blanks, reported in Figure 6, showed negligible weight losses until 300 °C, suggesting that all volatiles evaporated during autoclave consolidation of the blank. The TGA results excluded that the delaminations and porosities developed in blanks during IR heating could be associated with the evaporation of any volatile species.

**Figure 7:**
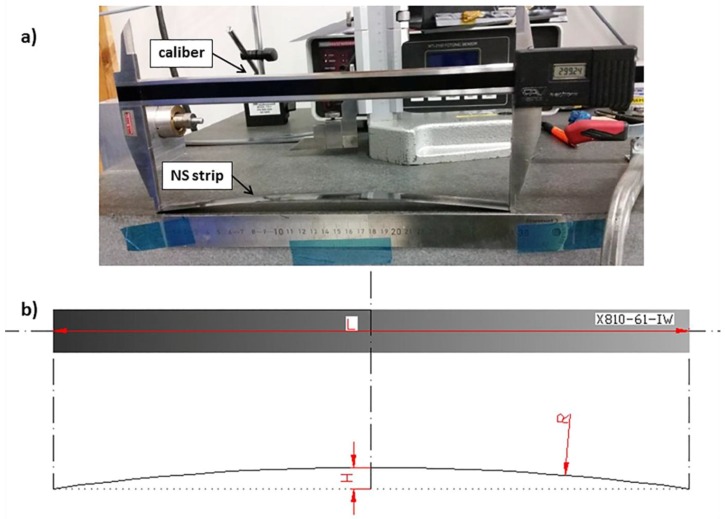
Deflection measurements for non-symmetric laminates [0, 0, 90].

Therefore, the residual internal stresses were considered the main cause of deconsolidation in blank laminates after IR heating [19]. An annealing treatment at a temperature above the glass transition temperature Tg of the matrix was proposed to allow the relaxation of the residual stresses generated during cooling in autoclave consolidation. The potential effectiveness of an annealing treatment at 240 °C, i.e., above Tg, for reducing the residual stresses was tested on non-symmetric laminates with the [0, 0, 90] stacking sequence, shown in Figure 7. The reason for the choice of this non-symmetric lay-up was due to the fact that the different shrinkage of the plies in directions 1 and 2 was responsible, during cooling, for the residual stresses leading to an easily measurable specimen curvature. The maximum deflection at the midspan, named H, of the non-symmetric strip reduced with increasing annealing time. In particular, the deflection H was dramatically reduced after an annealing cycle at 240 °C for 20 h, decreasing from 8.04 mm to 2.16 mm on a length of 30 mm, as reported in Table 2. This clearly indicated that the residual strain associated with the laminate configuration reduced as a consequence of stress relaxation phenomena occurring in the matrix dominate elastic properties, such as the transversal modulus and the interlaminar shear stiffness.

**Figure 8:**
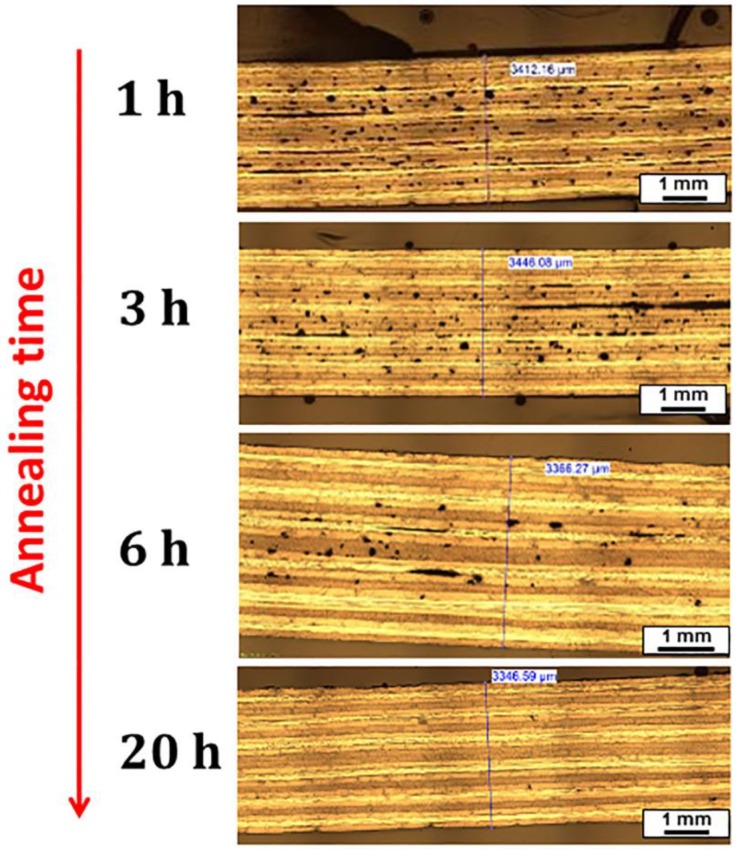
Optical microscopies of IR-heated blank laminates after annealing at 240 °C at different times.

The effect of different annealing times at 240 °C on reducing the porosity was then studied on IR-heated quasi-isotropic laminates. As observed by the optical microscopies reported in Figure 8, the annealed samples showed reduced porosities with the increase of the annealing time. After 20 h of annealing, porosities and delaminations were not detected anymore. These results were also confirmed by non-destructive evaluation carried out by ultrasound.

**Figure 9:**
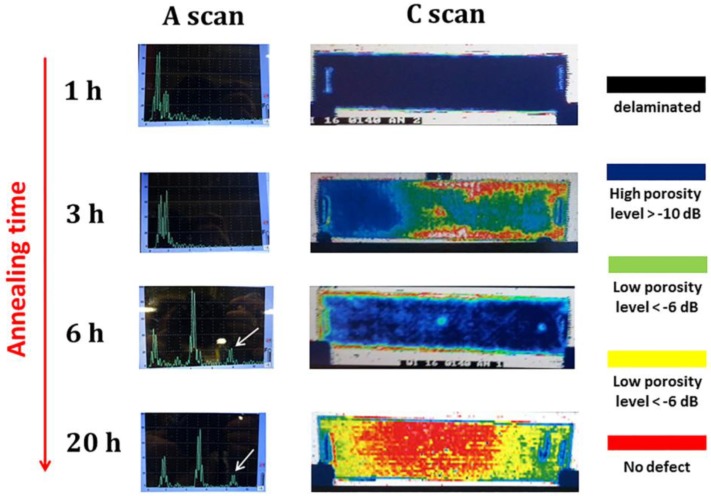
Non-destructive evaluation of blank laminates annealed at different times.

Non-destructive evaluation (NDE) was performed by means of A-scan and C-scan analysis. The A-scan presentation displayed the amount of received ultrasonic energy as a function of time of flight of the ultrasonic wave. In a pulse-echo mode detection, when the same ultrasonic transducer was used, either as a transmitter or as a receiver of ultrasonic waves, two echoes were visualized corresponding to the top and bottom interface of the examined sample. When the ultrasonic wave encountered a defect within the component, such as delaminations and porosities, the wave was reflected and scattered. Depending on the size of the defect, the back wall echo, corresponding to the bottom surface of the sample, was not detected due to the complete attenuation of the signal. As reported in Figure 9, A-scan images indicated that no back wall echo could be detected on IR-heated laminates for the large volume of defects until the annealing time reached 6 h.

The C-scan analysis, performed in transmission mode and reported in Figure 9, was a 2D image representation where the amplitude of the received ultrasonic wave signal acquired point-by-point on the laminate was used to map the component area [28]. From the C-scan image, complete delaminations were detected in as-received IR-heated and -dried samples (not shown for brevity) and samples annealed for 1 h (see Figure 9). Samples annealed for 3 h still presented delaminations and large porosities, while increasing the duration of the annealing treatment to 6 h led to a more uniform deconsolidation grade with small porosities detected by C-scan. The sample annealed 20 h at 240 °C presented no macroscopic defects, but still some zones, close to the laminate boundaries, were characterized by a small porosity within the acceptability limit for the production. The NDE analysis confirmed that the annealing treatment was able to dramatically reduce deconsolidation during IR heating, improving the chances of obtaining a press-formed part with reasonably low porosities and no delaminations.

Finally, the effect of drying and annealing on the quality of IR-heated quasi-isotropic blank laminates was studied by examining at the thickness changes before and after the heat treatments. The latter are reported in Table 3 as a function of the annealing time at 240 °C. The sample at time t = 0, even if dried 16 h at 150 °C, showed a significant change in the thickness (>7%), even if bulges were not observed from a visual inspection, as already shown in Figure 5. The thickness increase was inversely proportional to the annealing time at 240 °C reaching 0.45% after 20 h.

The exponential reduction of thickness changes (Δh) of blanks depended on the intensity of the frozen residual stresses accumulated during the cooling stage of autoclave consolidation. They were used to define a time-dependent «residual» strain ε_r_(t) proportional to these stresses:

ε_r_(t) = Δh/h_0_ (1)

roughly assuming a linear correspondence between these strains and stresses which relaxed during annealing.

**Figure 10:**
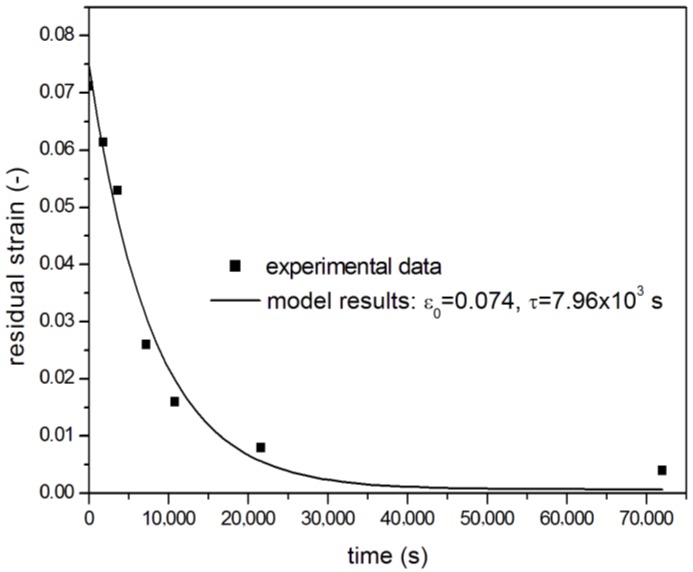
Residual strain of IR heated blank laminates as a function of annealing time at 240 °C, experimental data, and Equation (3) fitting.

Finally, a simple viscoelastic model was adopted to fit the data of Figure 10. Stress relaxation is usually modelled with several Maxwell models (spring and dashpot in series) in parallel, in order to account for several relaxation times. In this case, the measured data were not stresses, but thickness changes, which can be considered the direct consequence by the effect of buckling of the laminae of frozen residual stresses. Assuming that the lower the thickness increase, the lower the residual stress frozen in the laminate, it can be argued that the measured thickness h, can be used to obtain a residual strain ε_r_(t), depending on the annealing time, t:

ε_r_(t) = (h(t) − h_0)_/h_0_ (2)

where h_0_ was the initial thickness, measured before IR heating. This strain, in fact corresponded to the residual stresses developed during autoclave consolidation and relaxed during the annealing. The damper (matrix) had an infinite viscosity below the melting temperature. During IR heating, above the melting point, the matrix became a viscoelastic liquid, characterized either by an elastic modulus that is capable of deforming the laminate in the presence of residual stresses, either by a creep compliance allowing a strain in the laminate, leading to delaminations. Tensile stresses in the transversal direction led to compressive stresses along the adjacent 0° laminae, which buckled at high temperature when the matrix was liquid, generating bulges and delaminations.

Therefore, the degree of deconsolidation and the porosity content during IR heating depended on the intensity of the residual stress accumulated during cooling in the autoclave consolidation: these stresses were reduced during annealing above Tg, a temperature at which the matrix was characterized by a rubber-like behavior. For long annealing times, it was possible that the stresses decreased. If the residual stresses could be completely relaxed during the annealing, the residual deformation would not be observed during the infrared heating.

The data of Figure 10 were then fitted using a Maxwell model with a single relaxation time, τ:

ε_r_ = ε_0_ × exp(t/τ) (3)

The results of data fitting, shown in Figure 10 as a continuous line, indicated a good agreement between Equation (3) and experimentally-measured residual strains, mainly at low annealing times (t < 6 h), even using a single relaxation time. At longer times, a more complex model with two or more relaxation times could better fit the residual strain value. A relaxation time of about 8000 s was obtained.

## 4. Conclusions

In this work, the mechanisms of deconsolidation and defect formation in CF/PEKK composites produced by press-forming were analyzed. The defects in the final composite were associated to poor blank quality after infrared heating which was the consequence of residual stresses developed during autoclave consolidation of the blank laminate.

An approach to reduce deconsolidation and void content in heated blanks was proposed in order to minimize the defects in the final press formed product. A drying cycle below the Tg of the PEKK matrix limited the development of macroscopic surface defects of blanks after IR heating. However, this treatment caused no reduction of porosities in the structure. An annealing treatment at a temperature above the glass transition temperature of the matrix was able to dramatically reduce the residual stresses improving blank quality after IR heating. The stress relaxation phenomena during annealing were modeled using a simple viscoelastic model obtaining a relaxation time of about 8000 s.

## Figures and Tables

**Table 1:** Composite samples analyzed in this work and the characterization methods.

Samples	Stacking Sequence	Nominal Thickness (mm)	Characterization Methods
Quasi-isotropic (QI) blanks	[−45, 90, 45, 0]_3s_	3.3	TGA NDE Thickness
Non-symmetric (NS) strips	[0, 0, 90]	0.4	Curvature measurement

**Table 2:** Results of deflection measurements on non-symmetric strips after an annealing cycle at 240 °C for 20 h.

Non-Symmetric (NS) Strips [0, 0, 90]	L (mm)	H (mm)	R (mm)
Before Annealing	298.72	8.04	1391.35
After Annealing	299.23	2.16	5182.71

**Table 3:** Thickness changes measured on blank laminates after IR heating as a function of the annealing time at 240 °C.

Annealing Time at 240 °C (h)	Average Thickness Change (%)
0	7.67
0.5	6.15
1	5.35
2	2.67
3	1.63
6	0.83
20	0.45
